# Sensitivity Analysis and Influence Evaluation of Progressive Wall Thickness of Honeycomb Structures as Energy Absorber Produced by Additive Technology Multi-Jet Fusion

**DOI:** 10.3390/ma18010001

**Published:** 2024-12-24

**Authors:** Tomas Kalina, Zdenek Chval, Frantisek Sedlacek, Stanislav Spirk

**Affiliations:** Faculty of Mechanical Engineering, University of West Bohemia, 301 00 Pilsen, Czech Republic; zdchval@fst.zcu.cz (Z.C.); spirks@fst.zcu.cz (S.S.)

**Keywords:** cellular structures, honeycombs, lattice structures, energy absorber, explicit solver

## Abstract

The aim of this study was to investigate the potential of polymeric cell structures for the production of energy absorbers and to focus on the geometric optimization of polymeric cell structures producible by additive technologies to achieve the required deformation characteristics, high material efficiency and the low weight of the resulting absorber. A detailed analysis of different types of cell structures (different lattice structures and honeycombs) and their properties was performed. Honeycombs, which have been further examined in more detail, are best suited for absorbing large amounts of energy and high levels of material efficiency at known load directions. Honeycombs have the potential to absorb large amounts of energy relative to their low weight and their deformation characteristics have a relatively constant course. Honeycombs have the major disadvantage of an initial peak. However, this peak can be removed by appropriately adjusting the geometry of the honeycomb. Thanks to the possibilities that additive technology allows us, honeycombs with progressive wall thickness have been designed and researched. The output of this study is a detailed analysis of the properties and several design recommendations for the design of a honeycomb with a progressive wall thickness to achieve the required properties.

## 1. Introduction

Various cellular structures such as foams, honeycombs or lattice structures are most commonly used in practice to absorb mechanical energy. The mentioned structures deform elastically and plastically under the action of force or impact. They can be subjected to high compressive stress at a relatively constant stress value, resulting in a gradual absorption of energy and an even distribution of force on the protected object. This is used in practice, for example, if we want to protect something from damage (packaging technology), for the production of protective elements for people (protectors, helmets), or in the construction of deformation zones of means of transport.

The mentioned structures are mainly used for their very good ratio between absorbed energy, size and weight.

### 1.1. Cellular Structures

Specific materials and structures are chosen according to the specific application (anticipated direction and size of the load, built-in spaces, weight, price, or other additional requirements, such as resistance to weather effects, requirements for maximum deceleration, or the maximum force acting on the object under a given load, etc.).

Polymer and metal foams are very widely used energy absorbers because they are cheap, light and not too complicated to manufacture. There is a large number of types of foams and therefore a wide range of properties to choose from for specific applications. The disadvantage of foams is that they are stochastic [1], i.e., they have a randomly arranged microstructure. In large volume, however, the effect of randomness usually disappears and foams are usually isotropic from the perspective of the macro-model.

Since the micro-level structure of these materials plays a large role in their global mechanical properties [2,3], researchers have sought to find better alternatives for foams. Lattice structures produced by 3D printing can largely overcome this limitation.

In essence, lattice structures are very similar to foams with a carefully ordered and regular microstructure. Lattice structures are widely used as shell fillers in 3D printing. Thanks to the use of these structures, it is possible to save weight, material and time during production, while ensuring the very good rigidity and strength of the resulting product. However, lattice structures can also be used as energy absorbers and thus become a product in themselves. The concept of lattices and similar structures comes from retaining material only in the necessary regions of a part to attain a lightweight structure, while maintaining the global mechanical properties of parts [4]. By default, regular lattice structures generated in the pre-processor are used for the fillings of printed parts, but with the help of specialized FEA tools, even these structures can be optimized [5], similarly to topological optimization.

**Figure 1:**
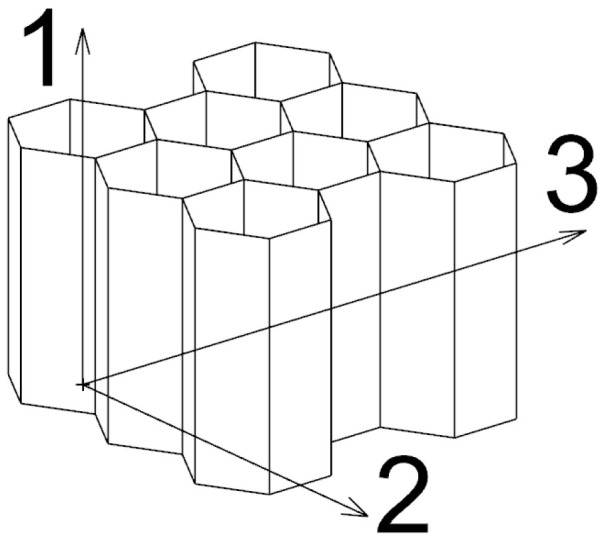
Basic honeycomb model.

Honeycombs are thin-walled structures usually consisting of regularly repeating equilateral hexagons. Honeycombs usually have orthotropic properties. In the direction perpendicular to the hexagonal profiles ((1) in Figure 1) they have significantly greater stiffness and strength than in the other two directions. Honeycombs are advantageously used in applications where the direction of the load can be predicted. In this article, the overall external dimensions of the honeycomb are considered in the direction of 1—height; 2—width; and 3—depth.

Honeycombs are usually made from thin sheets (from aluminum alloys, Kevlar, etc.). The basic geometric parameters of the honeycomb are cell size and sheet thickness. After choosing the basic geometric parameters, everything of course depends on the overall external dimensions of the body created from this honeycomb. With regard to the production technology of honeycombs [6], there is not much room for any shape optimization.

Additive manufacturing (AM) is one of the fastest growing industries today. Previously, this technology used to be called rapid prototyping; however, with the development of new technologies and materials, final parts (for small-scale production) and not just prototypes are increasingly produced using additive technologies [5].

Additive technologies allow us to create geometries that cannot be produced by other known production technologies. Examples can be precisely lattice structures, or geometrically complex cavities, or channels in forms.

The aim of this study is to explore the potential of polymer cellular structures for the production of energy absorbers and obtain a closer focus on the geometric optimization of cellular structures that can be produced by additive technologies to achieve the required deformation characteristics, high material efficiency and the low weight of the resulting absorber. The output of this study should be a comparison of which structures are suitable for absorbing a large amount of deformation energy and basic recommendations for their construction.

### 1.2. Three-Dimensional Printing Technology and Material

PA12 material printed on an HP Multi-Jet Fusion (MJF) 4200 printer (Hewlett-Packard, Palo Alto, CA, USA) was chosen for the investigation of printed cellular structures. There were several reasons for this choice. MJF technology is a significant innovation in the field of additive technologies after a long time.

**Figure 2:**
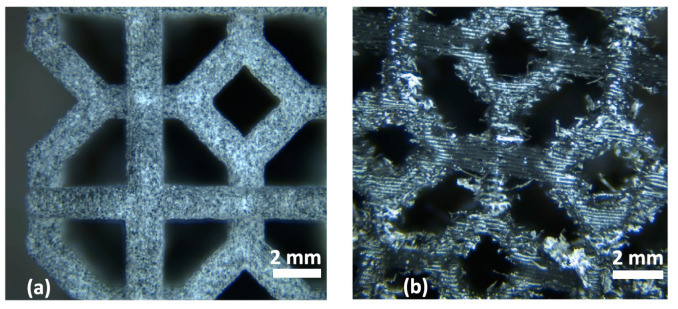
Detailed images of the structure printed by (**a**) MJF; and (**b**) FDM [10].

MJF technology brings significantly faster, but above all, more accurate printing than the previously known polymer printing technology. The layer thickness is 80 microns and the print resolution (in the horizontal plane) is of 1200 dpi, i.e., it has an accuracy of almost 20 microns. Figure 2a shows the detailed images of the structure printed by MJF technology, and Figure 2b shows a structure printed by conventional FDM (fused deposition modeling) technology. By comparison, we can observe that MJF achieves significantly better geometric accuracy and does not have visible print layers, as is the case with an equally large FDM sample [7,8,9].

**Figure 3:**
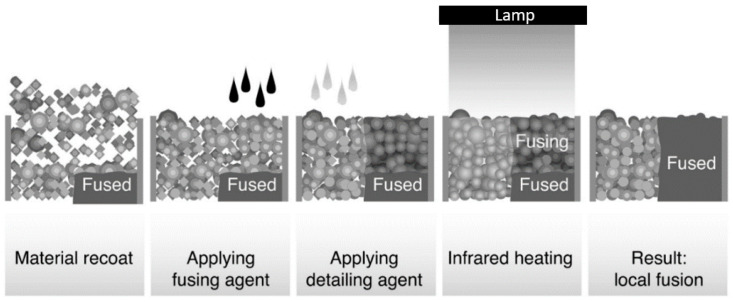
Multi-jet fusion process.

Like SLS (selective laser sintering), the MJF process uses thermoplastic powders to produce parts which carry similar mechanical properties to those of its SLS counterpart. The MJF technology, however, uses an ink-jet array to disperse a fusing agent as well as a detailing agent over the powder bed along with infrared (IR) lamps, instead of a laser. The ink-like fusing agent increases the absorption ratio of the IR energy, which is emitted over the powder bed, to be absorbed selectively, as shown below in Figure 3 [11,12].

The material PA12 is a thermoplastic with very good mechanical properties, suitable for the production of functional prototypes and final parts. The material properties are close to isotropic properties (the mechanical properties in the printing plane and perpendicular to this plane differ very little, in the order of units of percent, which is significantly less than in additive technologies known so far).

Both the material and the technology were chosen with regard to being able to distinguish very small nuances on the examined samples and to reduce the introduction of damage to the microstructure, as it is dealt with in [13].

**Figure 4:**
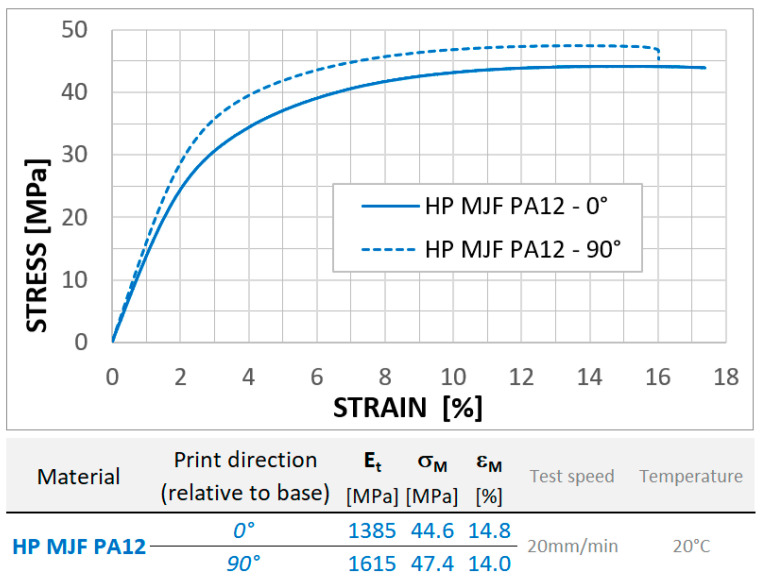
Mechanical parameters for HP PA12.

Figure 4 shows the material data that were measured at our workplace. We can see here that compared to common polymer-printed materials, the difference in measured properties in the (horizontal) print plane and perpendicular to the horizontal plane is relatively small. For example, the strength in these directions differs by only about 6%.

**Figure 5:**
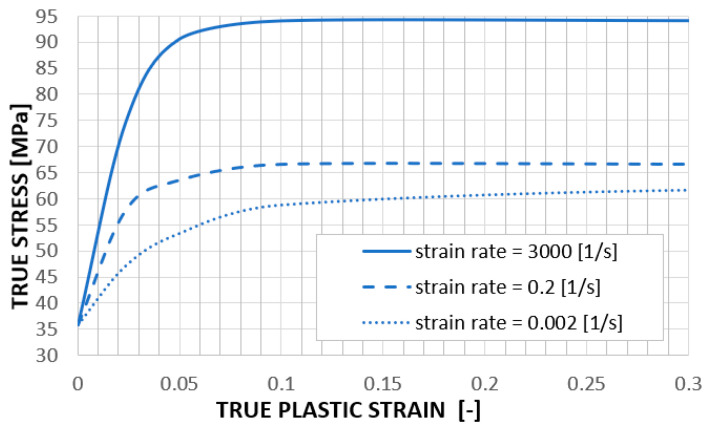
True stress dependence on true plastic strain for different strain rates.

**Figure 6:**
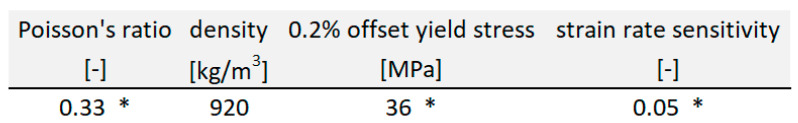
Other material properties of HP PA12 used in the FEA * [14].

Figure 5 shows the course of tensile tests at different strain rates. These data serve as an input to the numerical simulations. From these curves, the numerical solver approximates the values for the actual strain rate that occurs during the simulation. Figure 6 shows other material properties of HP PA12 used in the FEA.

MJF technology is the best that can be used to print such structures from polymers today.

### 1.3. Cellular Structures Made by Additive Technologies

Lattice structures (LSs) are typically created by repeating three-dimensional unit cells. There exists a vast array of lattice structures with a wide variety of geometric configurations. These structures offer the flexibility to engineer specific meso-scale properties (at the cell level), enabling the achievement of desired macro-scale material properties suitable for various engineering applications [14,15].

For instance, Linul et al. [16] demonstrated that the design of unit cell shapes is an effective approach to controlling the mechanical characteristics of reticulated meshes, such as elastic modulus, compressive strength and deformation behavior. Ashby [2] identified the classification of lattice structures into tension-dominated and bending-dominated types as a fundamental concept for analyzing their mechanical behavior. For a given density, tension-dominated structures exhibit high stiffness and strength, while bending-dominated structures are more flexible, with lower strength but superior energy absorption during compression.

Gautam et al. [17] investigated the effects of build orientation, truss radius and surface roughness on the stiffness, strength and energy absorption of ABS Kagome truss unit cells manufactured using fused deposition modeling (FDM). Kaur et al. [18] examined the deformation of two types of 3D-printed stretch-dominated micro-lattice structures made from different polymeric materials through compression tests and finite element analysis (FEA) simulations. Mohsenizadeh et al. [19] experimentally demonstrated that lightweight polymeric metamaterials for energy absorption can be designed and additively manufactured, and these materials are capable of recovering their original shape after significant deformation [14].

While most existing studies focus on the behavior of individual lattice structures with specific unit cell geometries, there are limited comparisons of the mechanical response and energy absorption characteristics of different types of 3D-printed lattice structures with identical relative densities [14]. Habib’s study [14] investigated how various 3D unit cell topologies affect the energy absorption and compressive response of 3D-printed polymeric lattice structures with uniform relative density.

In his next study [10], Habib compared several cell structures with honeycomb (HC). Habib’s numerical and experimental results show that when the relative density is kept constant, the out-of-plane energy absorption of the traditional honeycomb significantly outperforms examined lattices for all loading rates.

Of course, LSs have an advantage compared to HC, for example, in that they can have similar properties in all three directions, a larger STRAIN value can be achieved before the densification of the structure. LS, with its characteristics, is closer to the definition of an ideal energy absorber, which is explained in detail in Section 4.3 [10]. If high energy absorption and the total weight of the energy absorber are important to us, it is most advantageous to use HC, because the material is used more efficiently here, when it is deformed in a direction perpendicular to the cross-section plane of the profile.

Honeycomb have a relatively simple geometry with regard to their production technology [6].

**Figure 7:**
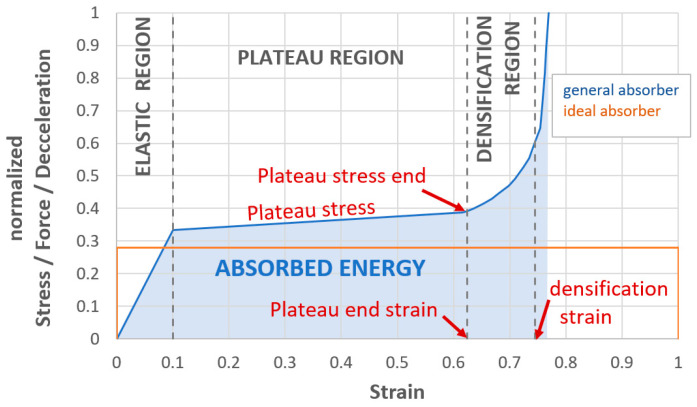
Idealized deformation characteristics for honeycombs, but also other lattice structures.

Figure 7 shows an idealized characteristic for honeycombs, but also other cellular structures (general absorber = blue-line). The characteristic consists of three basic areas. The first is the area of elasticity, where there is a gradual increase in the characteristic. The second area is the so-called “Plateau region” [14,20]. In this area, small parts of the structure gradually collapse (loss of stability). The deformed structure folds/breaks/cracks. Both elastic and plastic deformation occur here and the largest part of the energy is absorbed here. Honeycombs are at risk of buckling/loss of stability, which is described in [21].

In a typical cellular solid, as illustrated in Figure 7, the plateau region begins at the crush (yield) strain, *ε_y_*, marking the onset of a new deformation mechanism in the cell edges. This region continues until reaching the critical strain, *ε_cd_*, which signifies the end of the stress plateau or the beginning of densification. The plateau stress can be expressed using Equation (1) [14]. For a typical cellular solid, as shown in Figure 7, the plateau regime starts from the crush (yield) strain, *ε_y_*, representing the initiation of a new deformation mechanism of the cell edges, and ends at a critical strain, *ε_cd_*, representing the end of stress plateau or onset of densification. The plateau stress is given by Equation (1) [14].

(1) $$\sigma_{pl}=\frac{\int_{\varepsilon_{y}}^{\varepsilon_{cd}}\sigma \left(\varepsilon \right)d\varepsilon }{\varepsilon_{cd}-\varepsilon_{y}},$$

The last area is the densification region. In this part, the deformed structure settles and a large increase in pressure occurs. When protecting an object, we should not get into this area, because there is a huge increase in voltage, contact force and deceleration of the impactor very quickly, which has a negative effect on the protected object.

The denser the structure, the less strain we achieve. Lattice structures can reach strain values of even more than 0.9. Honeycombs usually range up to 0.8 strain. Lattice structures can thus be closer to an ideal energy absorber (orange-line in Figure 7) in which there would be the uniform absorption of energy from the beginning to strain 1.0.

As already mentioned, Figure 7 shows a simplified characteristic. The real characteristic usually has a pronounced initial peak, which is located at the end of the elastic region. The initial peak is caused by the initial resistance of the material on impact. Different materials and different structures show different initial peaks. Honeycombs, or lattice structures with rods stressed for buckling, usually have a significantly larger peak than lattice structures with rods stressed for bending (however, as already mentioned, these structures absorb significantly less energy for the same absorber weight).

If it is necessary to change the deformation characteristics of the honeycomb, which is usually achieved by layering different honeycombs on top of each other, and plates are inserted between the individual honeycombs, which distribute the load from one layer of the honeycomb to the other, and everything is connected using adhesives. Without the inserted plates, the honeycombs would cut into each other. However, the solution with plates brings a lot of added weight, and in addition, there are significant interfaces in the material as a whole. This additional material does not provide any increase in absorbable energy. Another way of modifying the characteristics of the honeycombs is creating holes in the honeycombs, or “shaping” them. However, this method is burdened by not very accurate production. Drilling cross holes weakens the honeycomb, but will reduce the weight only minimally (the material will remain trapped inside for the most part). Drilled holes are difficult to take into account in numerical simulations.

Additive technology, however, allows us to make sudden and continuous changes in the shape or thickness of honeycomb structures without any additional material. All the material used can thus participate in the absorption of deformation energy, i.e., we achieve greater “material efficiency”, less weight and a very precise geometric match between the virtual model and the actually manufactured part.

**Figure 8:**
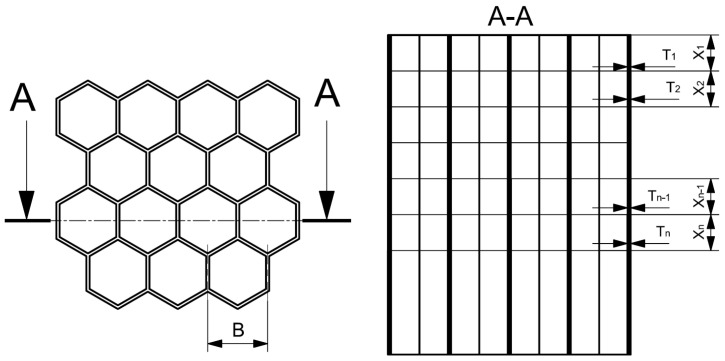
Model with graduated progressive wall thickness.

Among other things, the aim of this study is therefore to evaluate the influence of honeycomb with progressive wall thickness. With this approach, it should be possible to influence the deformation characteristics of the honeycomb, and mainly to reduce the initial peak. The principle of graduated progressive wall thickness is indicated in Figure 8.

It applies here that

*T*1 < *T*2 < *T*3 < … < *Tn* − 1 < *Tn*, (2)

and the default assumption is that

*X*1 = *X*2 = *X*3 = … = *Xn* − 1 = *Xn*. (3)

General evaluation of deformation characteristics of LS and HC.

Different polymer lattice structures were also tested in the past [22,23], but we reached the same conclusion as Habib [10,14]. Lattice structures achieve good material efficiency and evenly distribute the load on the protected object, or they absorb energy evenly, but compared to honeycombs, they absorb significantly less energy at the same relative density (that is, at the same weight). To achieve the same amount of energy, lattice structures would have to be significantly heavier and significantly bulkier than honeycombs. Therefore, lattice structures are unsuitable for the goals defined by us, but they are used in many other applications where they can apply their advantages. A detailed comparison of lattice structures and honeycombs is given in [10] and is not detailed here.

**Figure 9:**
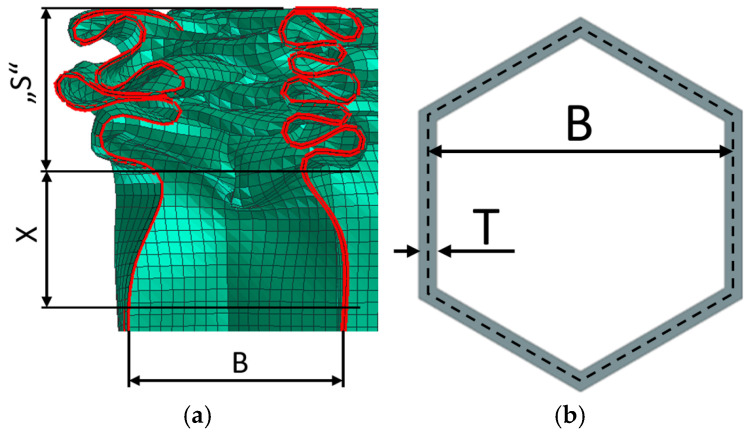
(**a**) Details of the folding of the honeycomb in the section, (**b**) definition of the dimensions of the honeycomb.

Only the honeycombs were further examined in detail. As already mentioned in the introduction, honeycombs have a very good ratio of absorbed energy to weight. Their only major drawback is that it is a buckling-stressed structure. Before the first loss of structure stability, the honeycombs show a distinct peak (see Figure 7, above). These peaks are usually undesirable. In the peak area, there is no significant increase in absorbed energy, but there is an increase in the force acting on the protected object, or a significant increase in the deceleration of the impactor, which in turn is undesirable in the case of the deformation zones of transport vehicles. Peaks in a very short time (units of milliseconds) may not always be critical; however, in general, it can be said that if peaks can be reduced or even eliminated, this is almost always a desirable benefit. After the initial peak, the deformation characteristic of the honeycombs is relatively constant. During the deformation of the honeycomb, the stability of a small layer of the structure (loaded for buckling) is lost, which gradually folds or breaks, and thus the deformation characteristic oscillates around a certain level (PLATEAU REGION—(see Figure 7, above)). Figure 9a shows the “S” region where the structure is already deformed (folded) and the “X” region, which is the region where there is an incipient loss of stability and this region will create another “fold/bend”. Figure 9b shows the basic dimensions of the honeycomb cell.

Note: Similarly, peaks are loaded lattice structures with buckling members (members parallel to the direction of loading). On the contrary, lattice structures with strips stressed mainly by bending have a smooth onset of deformation characteristics, without peaks.

Honeycombs and lattice structures with rods stressed for buckling have only a small proportion of elastic deformation, and the majority of energy is absorbed during the plastic deformation of the structure. Lattice structures with strips stressed mainly by bending show a significantly larger proportion of elastic deformation. This results in their suitability for applications where there is no need to absorb a large amount of energy, but on the contrary, it is desirable that a significant elastic deformation occurs even under a small load without breaking the structure.

## 2. Investigation of Dynamic Properties of Honeycombs Based on Experimental and Numerical Methods

### 2.1. Numerical Analyses

#### 2.1.1. Dynamic Phenomena in Numerical Simulations: Explicit Finite Element Analysis (FEA)

The explicit method is evidently a more suitable choice for the dynamic crushing of structures, as its time integration approach is well suited for processes that involve large deformations and significant shape transformations. Explicit solvers are generally preferred for problems characterized by complex contact conditions. Moreover, this approach significantly accelerates computations since it avoids the inversion of the stiffness matrix. In such scenarios, any nonlinearity is incorporated at the nodal force levels.

Explicit algorithms are fundamentally based on Newton’s second law of motion, represented as an equation in matrix form. This equation is defined for a specific moment in time. To achieve dynamic force equilibrium, the following expression must hold true [24,25].

(4) $$M\dot{v}=f^{ext}-f^{int},$$

Here, M represents the mass matrix, and $f^{ext}$ and $f^{int}$ are the nodal force matrices corresponding to the external forces and the internal resistance of the element. This relationship can be written in matrix form and is defined for a specific moment in time.

(5) $$\left\{F_{t}^{int}\right\}=\sum \left(\int_{\Omega }{\left[B\right]}^{T}\left\{\sigma_{n}\right\}d\Omega +\left\{F^{houg}\right\}\right)+\left\{F^{cnt}\right\},$$

Furthermore, $\left\{\sigma_{n}\right\}\;$represents the internal stress matrix, and $\left[B\right]$ is the matrix describing the shape change elements. The term $\left\{F^{houg}\right\}$ is included to mitigate the hourglass effect, while $\left\{F^{cnt}\right\}$ denotes the contact force vector. A key advantage of the explicit method is the use of elements with a single integration point, making it particularly suitable for large deformations and significantly reducing the computation time. In this approach, the energy and stress of the element are computed solely at a single integration point. However, this efficiency comes at the cost of reduced computational stability. The typical outcome of such computations is an imbalance between the kinetic and internal energy of the system, an issue referred to as hourglassing. Throughout the calculation, it is essential to continuously monitor the total energy, as an increase in hourglass energy beyond 5% of the system’s total energy is considered critical [24].

Explicit solvers are conditionally stable, with their stability primarily dependent on the size of the time step $t_{calc}$. This time step is governed by the propagation of stress waves through the material. The critical time step${\;t}_{crit}$ can be determined based on the time interval required for the stress wave front to traverse an element. Here, c is the velocity of stress wave propagation through the material, l is the characteristic dimension of the element, E is the modulus of elasticity, and ρ is the material density [24].

(6) $$t_{calc}\leq t_{crit}=\frac{l}{c}=l\sqrt{\frac{\rho }{E}},$$

#### 2.1.2. Pam-Crash: FEM-Based Computational Solver

Pam-Crash 18.0 is an FEM solver included in the VPS (virtual performance solution) software suite developed by ESI Group [26]. This software is designed for simulating impacts and evaluating passenger safety, with its primary application in the automotive industry. Its development began in 1978, coinciding with the first crash test simulations of automobiles. Based on the finite element method (FEM), Pam-Crash enables the modeling of complex geometries using a wide variety of element types. It supports an extensive range of linear and nonlinear materials, including elastic and visco-plastic materials, foams, multi-layer composites, as well as failure and defect models. Utilizing explicit FEM, it is well suited for nonlinear problems involving a large number of contacts, with numerous contact types available, primarily implemented through the penalty method [24].

The strain rate $\dot{\varepsilon }$ at the impact point can be estimated using the initial velocity of the striker and the initial height of the part:

(7) $$\dot{\varepsilon }\left(t\right)=\frac{d\varepsilon }{dt}=\frac{d}{dt}\left(\frac{L-L_{0}}{L_{0}}\right)=\frac{v\left(t\right)}{L_{0}},$$

Here, ε represents the engineering strain, $L_{0}$ is the initial width of the foam, L is the foam’s instantaneous width, and $v\left(t\right)$ denotes the instantaneous compression rate of the foam at time t.

#### 2.1.3. Computational Model for FEA

Numerical simulations were carried out using the FEM software Visual-Crash PAM 13.0 with an explicit solver [27,28] from the ESI Group, which is widely used among car manufacturers for simulations of vehicle crash tests. The honeycombs were modeled using 2D SHELL elements of the QUAD type with underintegrated elements [27] (with seven integration points through the thickness). The material was defined according to Figure 5 and the data in Section 2.

**Figure 10:**
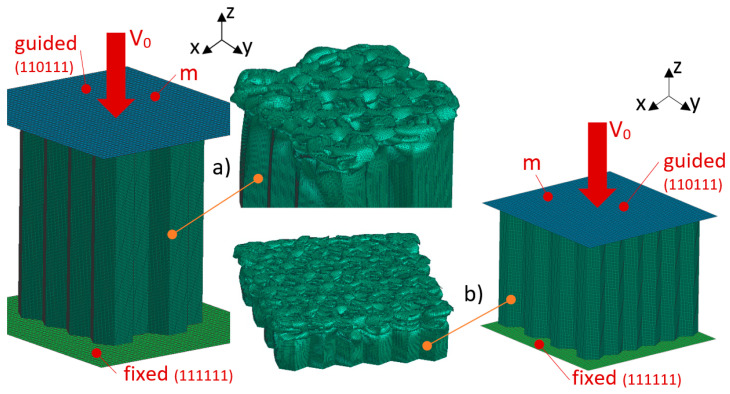
Calculation model and deformed honeycomb: (**a**) sample A and (**b**) sample D.

An impactor (rigid-body) of defined weight and defined pre-impact velocity hits the honeycomb. There are defined contacts between the honeycomb and the base and the ram. Self-contact is defined on the honeycomb. The computational model is shown in Figure 10.

### 2.2. Validation of Numerical Simulations According to Experimental Testing

**Figure 11:**
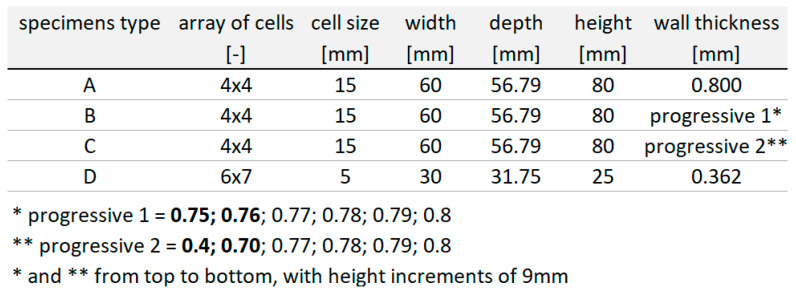
Geometric parameters of specimens.

On the basis of preliminary simulations, samples were defined for the validation of numerical simulations according to experimental testing. Four types of test specimens were defined (Figure 11). The two types of samples (A and D) are honeycombs with a constant wall thickness, each with a completely different height, size and number of cells. Samples A and D were loaded with significantly different parameters. The other two types of samples (B and C) are modifications of sample A. Samples B and C have, compared to sample A, a progressive wall thickness.

**Figure 12:**
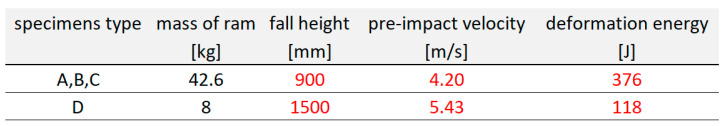
Impact parameters for individual specimens.

Type D specimens were tested on a smaller free fall drop tester and “A, B, C” specimens were tested on a larger free fall drop tester, so the test parameters are significantly different. The parameters of the experiments are shown in Figure 12. The samples were adapted to the available equipment.

#### 2.2.1. Experimental Testing

**Figure 13:**
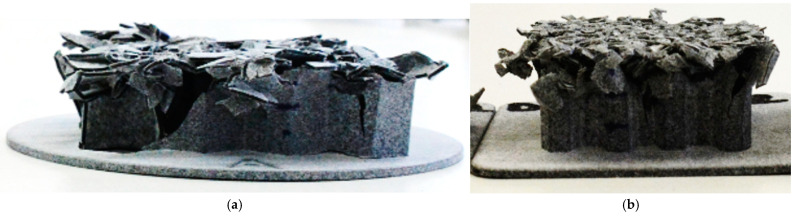
Specimens after experimental testing: (**a**) type A and (**b**) type D.

The experimental testing (Figure 13) was carried out on two different free fall drop testers with measuring equipment in the laboratories of Regional Technological Institute (RTI) and in the laboratories of the New Technologies for the Information Society (NTIS), which are research centers of the University of West Bohemia. A slow-motion camera, accelerometer and force cell were used for the measurement.

#### 2.2.2. Validation of Numerical Simulations

To validate the material models in numerical simulations, validation was performed on typical representatives of honeycomb samples.

D-type specimens, which have a very thin wall (which is almost borderline with the technology of upwelling and handling) achieve a very good agreement between the simulation and the experiment. Specimens of types A, B and C (with thicker walls) achieve solid results. The courses are similar, but the real specimens are a little more flexible than in the simulations.

**Figure 14:**
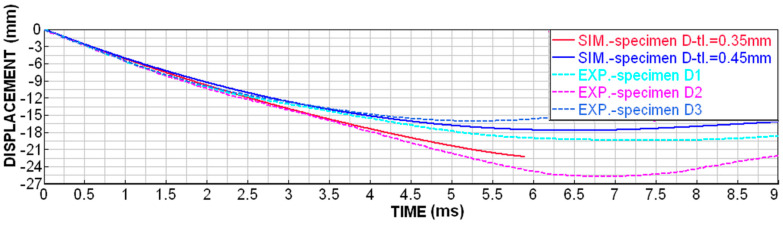
Displacement dependence on time for D-type specimens.

**Figure 15:**
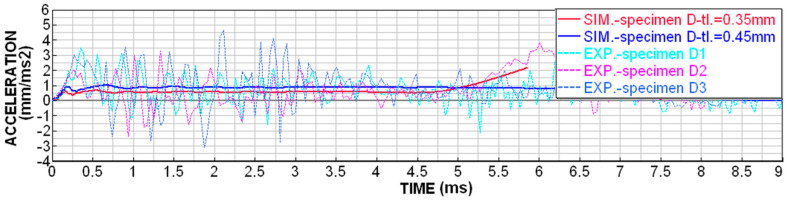
Acceleration dependence on time for D-type specimens.

**Figure 16:**
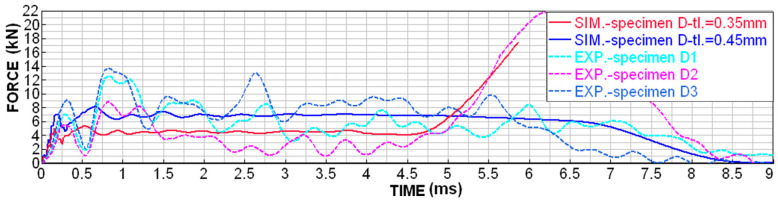
Contact force dependence on time for D-type specimens.

**Figure 17:**
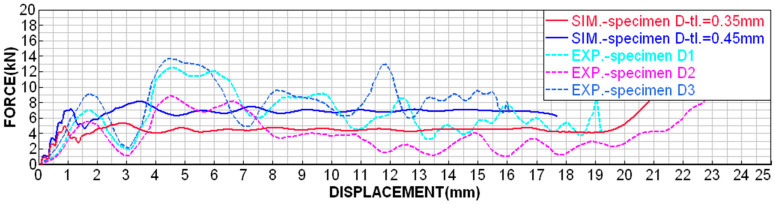
Contact force dependence on displacement for D-type specimens.

Although printing with MJF technology is very accurate, D-type specimens were very susceptible to uneven wall thickness due to the fact that the specimens are cleaned by sandblasting after printing. The sandblasting of a very fine structure results in the micro-wear of the surface, which is, however, noticeable with such thin walls (around 0.3 mm). For these specimens, it was possible to measure the wall thickness of around 0.35–0.45 mm. In order to be able to relevantly compare the numerical simulation with the experiments, both boundary conditions were simulated and compared, as shown in Figure 14, Figure 15, Figure 16 and Figure 17.

The dependence of the contact force on time shows higher values of peaks and dips in the experimental measurements (Figure 16), but the average level achieves a very good agreement.

Also, the dependence of contact force on displacement shows higher values of peaks and dips in experimental measurements (Figure 17), but the average level reaches a very good agreement.

**Figure 18:**
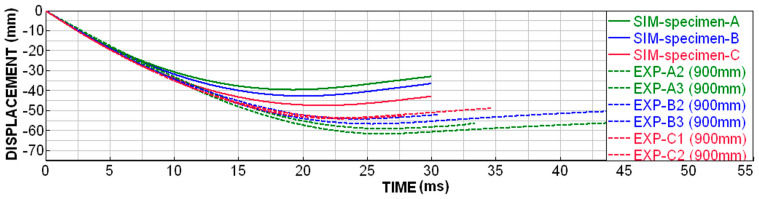
Displacement dependence on time for type A, B and C specimens.

For specimens of types A, B and C (with a wall thickness of 0.8 mm, with possible progression), it was possible to only compare the displacement in time (from the slow-motion camera). The courses from the numerical simulation and experiments are similar, but the real specimens are slightly more flexible than in the simulations (Figure 18). The higher flexibility of the specimens is caused by the fact that the real specimens partially break into larger fragments, which is not taken into account by the numerical simulation. The energy that would shatter these large pieces into smaller ones is missing here and is reflected in the fact that the specimens are more pliable in reality. Therefore, a higher height of the specimen must be deformed in order to capture the required amount of energy.

### 2.3. Evaluation of the Parameters Affecting the Properties of the Honeycomb

Many parameters will influence the deformation characteristics of a particular honeycomb. In particular, there are the material parameters and geometry. A simple honeycomb (cuboid shape) model can be described by several geometric parameters: total height, total width, total depth, cell size and wall thickness.

External (built-in) dimensions can often be given (either minimum or maximum). Their possible modification can significantly change the deformation characteristics of the honeycomb. All three dimensions have a great influence on the amount of energy that the honeycomb is able to absorb and on the course of the deformation characteristic.

If we consider the material, cell sizes and wall thicknesses as constant, then we can easily predict the effect of changes in parameters: height, width, depth or load changes on the resulting parameters: absorbed kinetic energy, deceleration and deformed height.

By increasing/decreasing the width, depth or height, we can increase/decrease the potential for energy absorption.

Changing the height of the honeycomb will not change the absorbed energy, deceleration, or deformed height, with the load unchanged. However, by increasing the height, the energy that the honeycomb is able to absorb will increase (therefore, however, a greater load needs to be developed).

Conversely, by changing the width or depth, the deceleration value and the deformed height will change, with the load unchanged.

Table 1 summarizes the basic knowledge of what affects what (namely how the results will be affected by changing the mentioned inputs). The results are always proportional to the general default solution. For example, if we increase the weight of the weight by two times, the energy will double, the deformed height will double and the average deceleration will be halved.

We can predict the above-mentioned influences (heights, depths, widths) relatively easily. However, the size of the cells and the thickness of the walls will also have an effect on the deformation characteristics of a particular honeycomb. And that even with a constant cross-sectional area, i.e., a constant volume, or constant weight of the honeycomb. However, we are not able to predict the influence of cell size or wall thickness so easily.

As for numerical simulations, the size of the mesh elements and the chosen time step can have a significant effect on the quality of the results.

All these unknowns were further investigated in detail using simulations and verified experimentally.

### 2.4. Sensitivity Analysis—Size of FEM Mesh Elements

**Figure 19:**
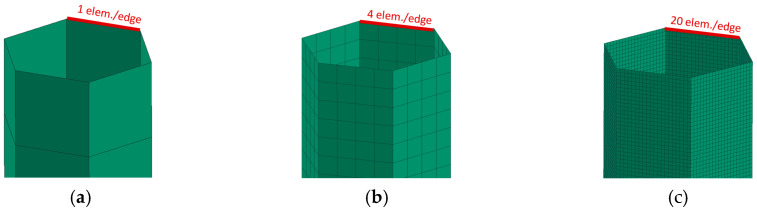
Calculation model, meshed with (**a**) 1 element, (**b**) 4 elements and (c) 20 elements per edge.

The size of the FEM elements of the mesh relative to the size of the part, or some of its parts (e.g., wall thickness, or the size of an edge), is a factor that can have great influence on the quality of FEA. If the size of the elements is clearly inappropriate, a large simulation error may occur, which will be diametrically different from the real behavior. For this sensitivity analysis, sample A was used, which was meshed with a range of different meshes. For the honeycomb, the edge of the hexagon that forms the honeycomb was chosen as the basic geometric element. Calculation models from 1 to 20 elements per edge were created (Figure 19). Specifically, these were 1, 2, 3, 4, 5, 6, 7, 10, 15 and 20 mm.

The time-step has an effect on the quality of the results in the simulation, and the explicit solver by default calculates the necessary time step itself according to the size of the smallest element [27]. To make the results comparable, a uniform time step was set for all simulations for the sensitivity analysis. A time step of 15 × 10^−5^ ms was chosen, which is the time step that the explicit solver determined for our model with the smallest elements, i.e., with the largest number of elements on the edge.

In real honeycombs, a gradual loss of stability and buckling of a small layer (height) of the honeycomb and a “folding” of the walls occur as standard, as indicated in Figure 9. Metal crash-boxes, for example, which are usually found in the deformation zones of cars, behave similarly.

**Figure 20:**
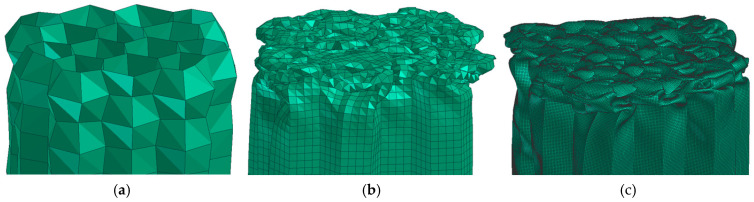
Sample folding examples with (**a**) 1 element to the edge, (**b**) 4 elements to the edge and (**c**) 20 elements to the edge.

Visually, the simulation with 4el is starting to get a little closer to this composition. to the edge in simulations. Models with 6 el./edge achieve better matches (in Figure 20). Visually nice folding occurs from 10 el/edge. With 20 el/edge, it occurs in “elegant” folding, like a metal box-beam. However, this is only a quick indicative assessment.

**Figure 21:**
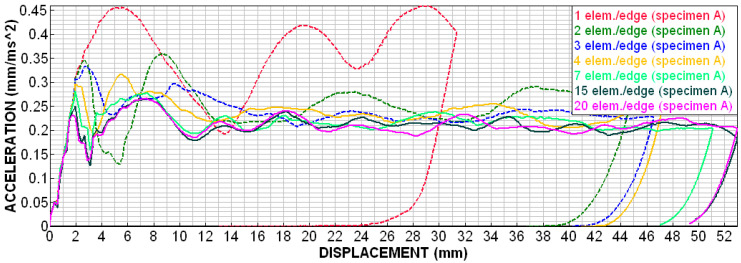
Sensitivity analysis—number of elements per edge, deceleration–dependence on displacement.

**Figure 22:**
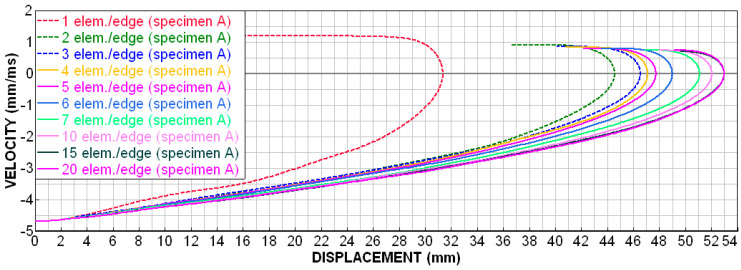
Sensitivity analysis—number of elements per edge, velocity dependence on displacement.

Figure 21 shows the deceleration–displacement dependences for selected models. We can see that there is no longer much difference between 7 el./edge and 15 el./edge, and the curves for 15 el./edge and 20 el./edge are already almost identical. A similar situation is depicted in Figure 22 (velocity–displacement). Again, the curves for 15 and 20 el./edge are practically identical, the curve for 7 el./edge does not differ much, and the curve for 1 el./edge is completely off (as expected). The conclusion from this sensitivity analysis is that for preliminary simulations, it is appropriate to use min. 4 el./edge, and for quality results, it is advisable to use min. 7 el./edge. The analyses in the upcoming chapters were performed with 15 el./edge for higher accuracy.

Models with 1 or 2 el./edge cannot achieve such a complexly curved shape as in Figure 9; therefore, these models visually, but also in terms of deformation characteristics, do not completely correspond with the real behavior of the honeycomb.

### 2.5. Sensitivity Analysis—Size of Cell Size

**Figure 23:**
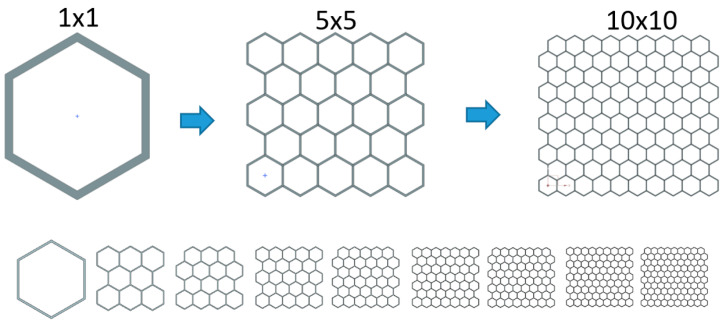
Examples of cross-sections of computational models of honeycombs.

An analysis of the sensitivity of honeycombs to cell size was performed. Sample A (4 × 4 cells) was chosen as the starting point, and it was subsequently modified. A series of computational models was created (in Figure 23), which always had dimensions of 60× approx. 60 mm, had an identical cross-sectional area and differed in the number of cells. According to the number of cells and uniform cross-sectional area, the wall thickness was calculated for each model.

**Figure 24:**
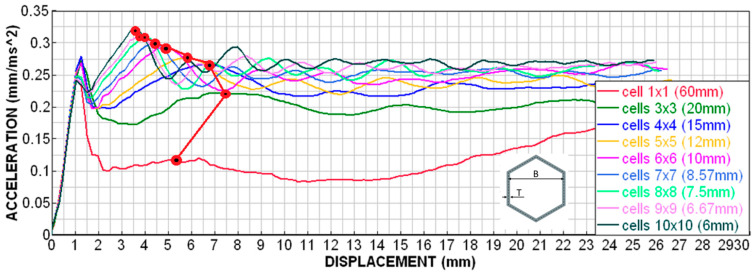
Sensitivity analysis—cell size—deceleration dependence on displacement.

From the given results (in Figure 24 and Table 1), we can see that, with the increasing number and decreasing size and thickness of the cell wall, there is a slight increase in the second deceleration peak and an increase in the total absorbed energy at uniform displacement. This is probably due to the fact that, the smaller the cells are, the more they are linked together, increasing the transverse stiffness of the honeycomb. We can observe a relatively smooth trend in the increase in the mentioned values. The only exception is the 1 × 1 sample, which is one giant element with very long and thin walls that tend to lose stability very easily.

### 2.6. Effect of Progressive Honeycomb Wall Thickness

**Figure 25:**
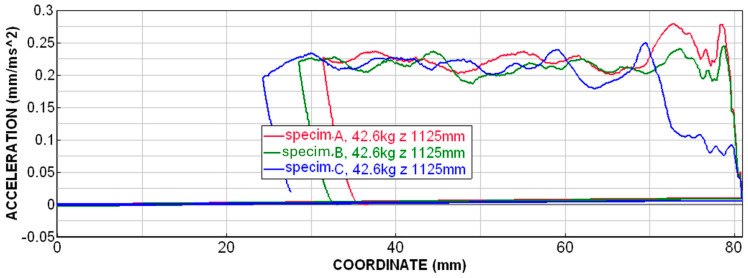
Deceleration dependence on displacement—specimens A, B and C.

In Figure 25, the deceleration courses are shown for specimens A, B and C. We can observe that specimen A (with a uniform wall thickness of 0.8 mm) has two significant peaks and then an oscillation around a steady level occurs. Specimen B has a gradual increase in thickness in its walls from 0.74 mm to 0.80 mm in steps of 0.01 mm. For this sample, it was possible to reduce the initial peaks to almost the level of average deceleration. The sample C has a gradation of the walls’ thickness 0.40; 0.70; 0.77; 0.78; 0.79; and 0.80 mm. We can observe there that a large gradation of the wall thickness only leads to a delay of the initial peaks and, on the contrary, can have a negative effect on their maximum value. The maximum peak of the specimen C is located between the maximum peaks of specimens A and B. Since the same energy must be absorbed during the impact, deformations occur on a larger path (larger specimen height) when the peaks are reduced or moved away.

## 3. Summarization and Discussion of Results

Honeycombs have great energy absorption potential compared to lattice structures, especially lattice structures with rods stressed in bending. The dominant part of the deformation characteristics of the honeycombs has a relatively constant course (Figure 7). However, the fundamental disadvantage of honeycombs is a pronounced initial peak which is caused by the initial resistance of the material on impact.

The initial peak can be influenced, for example, by modifying the external geometry of the honeycomb, or by intentionally disrupting the stability of the honeycomb, or by layering different types of honeycomb. However, all these methods are complex, imprecise, or lead to a reduction in material efficiency.

The use of the progressive thickness of the honeycomb wall appeared to be a suitable solution in combination with additive technology. In this way, a significant reduction in the initial peak was achieved, i.e., a gradual onset of the deformation characteristic was achieved. However, a high level of material efficiency was maintained.

A large number of analyses and sensitivity analyses were carried out in order to be able to define the basic knowledge for the design of honeycombs with progressive wall thickness produced by additive technology.

The effect of height, width and depth (overall external dimensions of the honeycomb) can be predicted relatively easily (on the basis of generally applicable rules and basic physical relationships). By increasing/decreasing the width, depth or height, we can increase/decrease the potential for energy absorption). Changing the height of the honeycomb will not change the absorbed energy, deceleration or deformed height, with the load unchanged. However, increasing the height will increase the energy that the honeycomb is able to absorb (therefore, it is necessary to develop a greater load). Conversely, by changing the width or depth, the deceleration value and the deformed height will change, with the load unchanged. Table 1 summarizes the basic findings on the influence of the overall height, width and depth of the honeycomb.

However, the size of the cells and the thickness of the walls will also influence the deformation characteristics of a particular honeycomb as well as even a constant cross-sectional area, i.e., a constant volume, or constant weight of the honeycomb. However, we are not able to predict the influence of cell size or wall thickness so easily. As shown in Table 2, with the increasing refinement of the structure (increasing the number of cells and decreasing the wall thickness), there is a slight increase in absorbed energy and deceleration.

As for numerical simulations, the size of the mesh elements and the chosen time step can have a significant effect on the quality of the results. All these unknowns were further investigated in detail using simulations and verified experimentally. The conclusion from this sensitivity analysis is that, for preliminary simulations, it is appropriate to use a minimum of 4 el./edge, whilst for quality results, it is advisable to use minimum 7 el./edge. For the best results, it is advisable to use approximately 15 el./edge.

For example, it has been found that, if we design a honeycomb with graduated progressive wall thickness, then it is appropriate to choose the “thickness graduation segment height” (X) (in Figure 9) in the range of 0.6 to 1.0 times the cell size (B), i.e., X = (0.6 to 1.0) * B. This finding results from how the honeycomb structure is folded. A segment of height (X) creates one loop in the folded structure (Figure 9).

In connection with the gradation of thickness, it was further found that too large steps between wall thicknesses do not bring the desired effect. During the transition between walls with a large difference in wall thickness, a similar effect occurs as during the initial impact on the honeycomb and this results in a significant peak. A significantly more pliable segment (with a significantly thinner wall thickness) will not sufficiently influence and prepare the next segment for deformation. In this way, we only delay the maximum peak, but this does not result in a significant reduction in the maximum peak. (Example of inappropriate grading: 50%, 100% of the max. wall thickness, or 10%, 30%, 50%, 70%, 100% of the max. wall thickness).

It is significantly more efficient to choose the thickness graduations in very small steps. There is no effect, as mentioned above, because the deformed segment affects the following segment, which thus “prepares” for deformation (it undergoes elastic deformation and a partial loss of stability).

If we know the nominal (maximum) wall thickness of the honeycomb with which we are to work, as well as the initial (minimum) wall thickness, it is advisable to choose a wall thickness whose maximum peak of the observed parameter (force, deceleration, etc.) is lower than the average value of the observed parameter for the nominal thickness.

In general, it can be said that the finest gradation is suitable (which can realistically be achieved with regard to the accuracy of production, possibly also the total height of the honeycomb) with the initial thickness. walls ≤ 90% of nominal wall thickness. (Example of appropriate grading: 88%, 91%, 94%, 97%, 100% of the maximum wall thickness.)

From the comparison of samples with the same specific density and the same external dimensions, it follows that it is advisable to choose a larger number of smaller cells with a thinner wall thickness. Although the compared samples have the same weight, a different distribution of the material leads to a slight increase in absorbed energy, or average deceleration, or average contact force at a specific deformed height. The reason for this phenomenon may be the greater interconnectedness of cells and thus their greater stiffness and the greater work required to deform the structure. The only limitation here are the technological possibilities of production (how big the part is (total external dimensions), so that we are able to print such a part and possibly clean it in the case of MJF technology.

The aforementioned smaller wall thickness has a further favorable effect on the folding and not splitting of the structure. When shredding material, attendance may chip off a large piece of material that will not be used effectively (no work will be consumed to shred it into small pieces). From this point of view, it is more advantageous when the structure is folded or broken into small elements.

The suitable size of FEM mesh elements for preliminary simulations, it is advisable to use a minimum 4 el./edge, for quality results, it is advisable to use minimum 7 el./edge. However, for a detailed comparison of different structures, it is advisable to use a minimum 15 el./edge, when the “elegant” folding of the structure occurs.

Recommendations for further research:

In the future, it would be beneficial to carry out a sensitivity analysis of the elasticity of thin rods (walls), on which it would be comparatively investigated to what extent they can be bent by 180° without the appearance of cracks. With respect to what angle it can be bent, without cracking, all this is considered with regard to the advantages of folding the structure against the unwanted breaking into large fragments.

## 4. Conclusions

A comparison of lattice structures and honeycombs was made, with a greater focus on honeycombs.

A detailed analysis of the deformation characteristics of honeycombs and an analysis of the influence of individual honeycomb parameters on the results of simulations and experimental tests was carried out, which can be used in the design of specific honeycombs. In general, however, it can be said that it is most effective to use honeycombs with the smallest possible cell size, but this has its limits given the production technology of the given honeycomb. When scaling the wall thickness, it is better to perform fine scaling (e.g., 88%, 91%, 94%, 97%, 100%), because the peaks of the characteristics are better eliminated.

Using a series of sensitivity analyses, recommendations were made for the parameters of the honeycomb simulations. In particular, this means the recommendation of the number of elements per edge.

The benefit of grading the thickness of the honeycomb wall was demonstrated and general recommendations were given for the design of grading in terms of both the thickness and height of the grading.

## Figures and Tables

**Table 1:** Dependencies between selected input and output parameters.

INPUTS							OUTPUTS			
Material	Geometric Parameters of Honeycomb				Load					
	Width/Depth	Height	B	T	Mass	Velocity	E	a_AVERAGE_	H_def._	A
def.	def.	def.	def.	def.	def.	def.	def.	def.	def.	def.
def.	**X-times** def.	def.	def.	def.	def.	def.	def.	**X-times** def.	**(1/X)-times** def.	**X-times** def.
def.	**X-times** def. **Y-times** def.	def.	def.	def.	def.	def.	def.	**(X ∗ Y)-times** def.	**(1/(X ∗ Y))-times** def.	**(X ∗ Y)-times** def.
def.	def.	def.	def.	def.	**X-times** def.	def.	**X-times** def.	**(1/X)-times** def.	**X-times** def.	def.
def.	**X-times** def.	def.	def.	def.	**X-times** def.	def.	**X-times** def.	def.	def.	**X-times** def.
def.	def.	**X-times** def.	def.	def.	def.	def.	def.	def.	def.	def.
def.	def.	def.	def.	def.	def.	**X-times** def.	**(X^2^)-times** def.	def.	def.	def.

Where: def. is default value, B is cell size, T is wall thickness, A is cross-section area, E is absorbed kinetic energy, a_AVERAGE_ is average deceleration, H_def_ is deformed height, Y and X are variables (from a set of real numbers). Assumptions: Specimens are always highenough, that means that densification of deformed structures can not happen. Wall thickness is significantly smaller that the cell size (t << B).

**Table 2:** Sensitivity analysis—cell size—geometric parameters of specimens and compared results.

Array of Cells [-]	Cell Size [mm]	Model Width [mm]	Model Depth [mm]	Wall Thickness [mm]	Absorbed Energy * [J]	decc. _MAX_ [m/s^2^]	decc. _AVERAGE_ * [m/s^2^]
1 × 1	60	60	69.28	1.832	124	243	119
3 × 3	20	60	57.74	0.956	210	272	199
**4 × 4**	**15**	**60**	**56.79**	**0.800**	**241**	**279**	**227**
5 × 5	12	60	55.43	0.637	249	277	236
6 × 6	10	60	54.84	0.557	260	292	249
7 × 7	8.57	60	54.43	0.477	262	300	251
8 × 8	7.50	60	54.13	0.429	269	310	257
9 × 9	6.67	60	53.91	0.381	272	310	262
10 × 10	6	60	53.69	0.349	279	320	268

* on a uniform displacement (deformed height) = 25 mm. uniform cross-sectional area = 380.7 mm^2^.

## Data Availability

The data presented in this study are available on request from the corresponding author. The data are not publicly available due to privacy and due to the complexity of this data.
